# COVID19 and the Media Introductory Note

**DOI:** 10.1177/2056305120947663

**Published:** 2020-08-10

**Authors:** Adrienne Russell, Matt Powers

**Affiliations:** University of Washington, USA

**Keywords:** COVID19, Media, 2K

## Abstract

All of the authors who have contributed to this issue on COVID19 and the Media have stepped out of the rush of change, if only for a 2000-word moment, to look into a corner of the mediated pandemic and grapple with the significance of what’s happening in our field, our societies, our lives. We appreciate their insights, and hope you do too.

In late-June 2020, authorities reported nearly 10 million confirmed cases of COVID19 worldwide and a half million deaths tied to the disease. The pandemic has fueled enormous individual loss and pain, and it has generated intersecting and large-scale economic, social, and political shocks. Loved ones died in isolation. Entire towns were quarantined. Jobs disappeared. Mental health eroded. Everyday life takes on bizarre contours. We wear face masks, homeschool, avoid neighbors, and Zoom-work, if we’re lucky. Indeed, we’re *living virtually*, an expression based in digital media technology that suddenly feels like it applies more broadly to our lives. How many aspects of these COVID19 lives we’re living will outlast the disease? The contributors to this issue of 2K take up that question in their own ways, exploring how media are shaping and being shaped by our new pandemic realities.

One of the most prominent media stories of the COVID19 era is the information crisis that has marked it. Pandemic news seems to come as fast as our quarantine days go slow. There is simultaneously a flood of misinformation and disinformation as well as a lack of vital information. Merrill Brown, founder and CEO of The News Project, said in June that the health tragedy has been compounded by an “information tragedy.”^1^ He was reacting to a US Center for Disease Control admission that the number of COVID19 infections could be 10 times higher than the 2.4 million reported cases. “News that 20 million people may have the virus merits a nationally televised White House briefing, not a call,” he said. Brown added that the United States “has no regular public briefings and no national spokesperson sharing government data or advice,” that the British government was “ending its daily Downing Street press conference, which 80 percent of the British say informs them,” and that in April, as cases were set to spike in Florida, that state’s governor, Ron DeSantis, told residents that he could see “light at the end of the tunnel.” The depth of the information crisis was already coming clear in February to Tedros Adhanom Ghebreyesus, director-general of the World Health Organization. He told a gathering of foreign policy and security experts in Munich that the organization was battling an epidemic as well as an “infodemic.” He said that untruths about the virus behind the pandemic were spreading “faster and more easily than this virus” itself.

Contributors to the issue take up various aspects of these developments—laws enacted in different countries in response (Radu, this issue), ways some religious groups are fueling the spread of misinformation (Alimardani & Elswah, this issue), the role played by YouTube and global messaging services such as WhatsApp and Telegram as vectors of misinformation (Malhotra, this issue; Marchal et al., this issue), the particular vulnerabilities of certain segments of the population to the spread of disinformation (Moore, this issue), and the way news organizations, hit hard by ad-revenue losses during the pandemic, have struggled to vigorously counter misinformation (Aronczyk, this issue).

Other contributors address the way the pandemic has spotlighted and exacerbated inequalities and encroachments on rights, for example, examining the fact that not all populations use digital media equally to create social connection, which may matter a great deal in light of the impact the pandemic has had on people’s mental health and well-being (Nguyen et al., this issue). Others focus specifically on the role of data collection and use, exploring ramifications of the fact that much data collection overlooks the global south (Trere & Milan, this issue), considering how the pandemic has lifted safeguards erected to protect human rights (Madianou, this issue), as well as the implications of mass surveillance both as a tool to combat the virus and as one that promotes inequity and undermines privacy (Frith et al., this issue).

In her reflections on the ways in which social media and data are implicated in emergencies, Mirca Madianou observes a shift in how technology is popularly framed: from a “source of addiction” to a “lifesaver,” a shift she argues, “which parallels previous oscillations in the field of media studies with pendulum swings between approaches that favoured either ‘strong media’ or ‘powerful users.’” Several essays here present a nuanced view of the tension between the power of the user and the powerful shaping of the larger political and technological contexts. They describe narrative tugs of war between publics and governments (Feng, this issue), the power of artistic expression and irony (Vicari & Murru, this issue), online community shaming as extemporized public health safeguard (Turkoglu & Odabas, this issue), efforts to combat pandemic-fueled xenophobia (Abidin & Zeng, this issue), and attempts by social media companies to update their content moderation practices to meet the reality of what the United Nations has called a mental health emergency (Gerrard, this issue).

In keeping with the goals of 2K to provide a space for scholars to reflect publicly about their research, several of the essays include explorations of methodological and conceptual challenges posed by the kind of logistical and existential jolts and transformations the pandemic is delivering daily (Hargittai et al., this issue; Kligler-Vilenchik et al., this issue; Kunelius, this issue). All of the authors who have contributed to the issue have stepped out of the rush of change, if only for a 2000-word moment, to look into a corner of the mediated pandemic and grapple with the significance of what’s happening in our field, our societies, our lives. We appreciate their insights, and hope you do too.

## Notes

1. See CNN (2020).
